# Research Progress on the Application of Biomass Fibers in Lithium-Ion Battery Separators

**DOI:** 10.3390/membranes15120361

**Published:** 2025-11-28

**Authors:** Chi Chen, Boqiao Li, Chong Zhao

**Affiliations:** 1 School of Chemistry and Environmental Engineering, Wuhan Polytechnic University, Wuhan 430023, China; x3466982687@163.com; 2 Hubei Key Laboratory of Agricultural Waste Resource Utilization, School of Chemical and Environmental Engineering, Wuhan Polytechnic University, Wuhan 430023, China

**Keywords:** lithium-ion batteries, biomass, fiber, separator, renewable

## Abstract

The separator is a key component of lithium-ion batteries, and its properties play a crucial role in the performance of such batteries. However, the most widely used polyolefin separators are not only made from non-renewable resources such as petroleum, but also have poor wettability to electrolytes, and their low melting points may cause short circuits or even explosions. Therefore, advanced separators that meet the increasing requirements of such batteries are urgently needed. Compared to polyolefin separators, renewable biomass fiber-based separators have better compatibility with electrolytes, higher thermal stability, and are naturally abundant. Their use is not only in line with sustainable development, but it also lowers their material cost. Therefore, biomass fiber-based separators are considered a promising candidate for replacing polyolefin separators for lithium-ion batteries in the future. In this article, studies on the preparation and application of biomass fiber-based separators in lithium-ion batteries in recent years are reviewed, looking forward to their future development, with the aim of providing a reference for researchers.

## 1. Introduction

**Figure 1:**
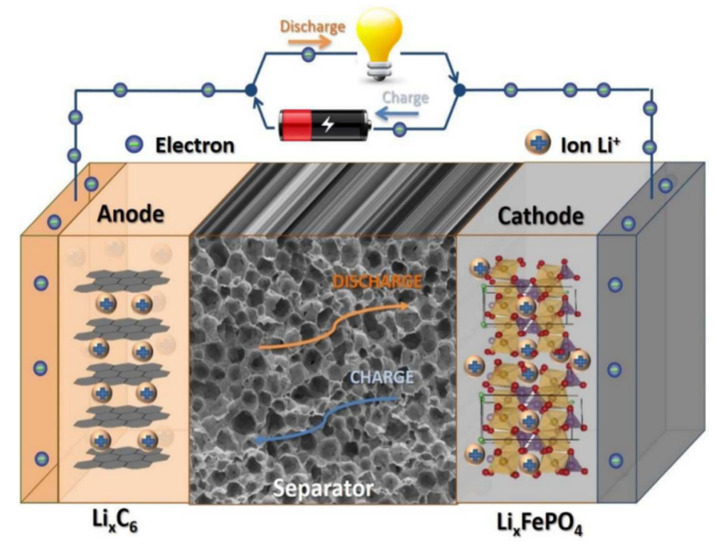
Schematic diagram of the main components of a lithium-ion battery [9].

Lithium-ion batteries have become ideal energy storage devices due to their high specific energy, high power, excellent charge and discharge cycling capabilities, and environmental friendliness [1,2,3]. Recently, they have been widely applied in portable electronic devices such as computers and cameras, as well as in large medical, automotive, and aerospace devices, and in other fields [4,5]. They are composed of mainly five parts: a positive electrode, a separator, a negative electrode, an electrolyte, and a battery shell [6]. The separator in lithium-ion batteries is a permeable membrane that physically isolates the positive and negative electrodes [7,8]. Its main function is to prevent short circuits caused by contact between the positive and negative electrodes, while allowing lithium ions to pass through and form ion currents, as shown in Figure 1 [9]. Therefore, the separator plays a crucial role in liquid electrolyte batteries [10,11,12]. Its electrolyte wettability guarantees efficient electrochemical reactions in lithium-ion batteries, and its pore structure (related to the transport rate of lithium ions), heat-resistant quality, and mechanical strength also significantly affect the energy density, safety, and lifespan of lithium-ion batteries [13,14]. However, the most widely used polyolefin (polyethylene (PE), polypropylene (PP), and their copolymers) separators have inherent hydrophobicity and relatively poor porosity, which has raised serious concerns over the insufficient wettability of the electrolyte, and their low melting points may cause short circuits or even explosions in lithium-ion batteries [15,16]. In addition, polyolefin separators are made from non-renewable resources such as petroleum, which are unsustainable and non-biodegradable [17,18,19]. Therefore, developing more advanced separators has become an urgent requirement for the development of high-performance lithium-ion batteries [20,21,22].

In recent years, widely sourced and renewable biomass fiber-based separators have attracted people’s attention and quickly become a research hotspot [23,24,25]. So far, a variety of biomass fiber-based separators, including pure biomass fiber-based separators and biomass fiber-based composite separators, e.g., cellulose/silica (SiO_2_), cellulose/polydopamine, cellulose/polysulfonamide, lignocellulose/polyvinyl alcohol (PVA), and polyformaldehyde/cellulose nanofibers (POM/CNF), have been fabricated and employed in lithium-ion batteries [26,27,28,29,30,31,32,33,34,35]. In comparison with traditional polyolefin separators, biomass fiber-based separators exhibit remarkably improved electrolyte absorption, heat resistance, and electrochemical properties, especially under high-temperature conditions [36,37,38,39,40,41,42,43,44,45]. Meanwhile, several approaches for manufacturing biomass fiber-based separators have been proposed and studied, such as electrospinning, forcespinning, vacuum filtration, spray deposition, and papermaking [46,47,48,49,50,51,52]. However, the immature large-scale production, such as the time-consuming process, still limits the practical application of biomass fiber-based separators [53,54,55]. In this article, the preparation and application of biomass microfiber-based separators, biomass nanofiber-based separators, and composite separators prepared by combining traditional polyolefin with biomass fibers in lithium-ion batteries are reviewed, with the aim of providing a reference for researchers.

## 2. Source and Characteristics of Biomass Fiber-Based Separators

Biomass fibers come from a variety of organisms such as plants (wood, bamboo, cotton, linen, etc.), animals (shrimp shells, crab shells, etc.), microorganisms (bacteria, fungi, etc.), and algae [56]. As the most widely available raw material for biomass fibers, cellulose accounts for (percent by weight) approximately 33% in vegetables, 40% to 50% in wood, and as high as 90% in cotton fibers. According to a rough estimate, cellulose represents an annual output of 150 billion tons of biomass, which makes biomass fibers a vast repository of renewable resources [53]. Compared to traditional commercial polyolefin separators, biomass fiber-based separators have several advantages [53,57,58,59,60]: (i) Biomass fiber-based separators have high porosity and are easily functionalized, providing tailored functionalities for the resulting compounds. (ii) Due to their hydrophilicity, the compatibility between biomass fiber-based separators and electrolytes is stronger, which is beneficial for electrolyte absorption in separators, enhancing the transmission efficiency of lithium ions and reducing the internal resistance in lithium-ion batteries. (iii) Biomass fiber-based separators have high thermal resistance up to 200 °C, while commercial polyolefin separators undergo thermal shrinkage above 90 °C and gradually melt above 150 °C. (iv) Unlike polyolefin substances produced from finite fossil oil, biomass cellulose is naturally abundant, biodegradable, and renewable. This is not only in line with the sustainable development of the environment but also greatly reduces the material cost of separators. Therefore, biomass fiber-based separators have high potential to replace polyolefin separators in lithium-ion batteries in the future.

## 3. Preparation and Application of Biomass Fiber-Based Separators

### 3.1. Biomass Microfiber-Based Separators

**Figure 2:**
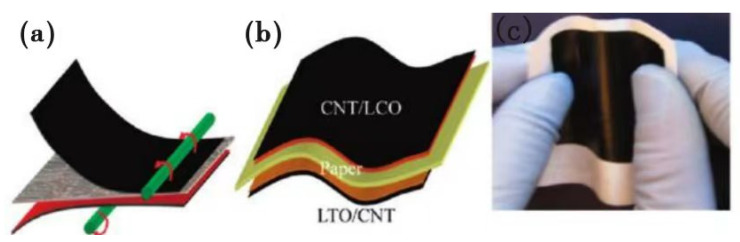
(**a**) Schematic diagram of the lamination process. (**b**) Schematic diagram of the lithium-ion paper battery structure. (**c**) Photograph of the lithium-ion paper battery before packaging [61].

Biomass microfibers are one of the most important components of plants; they are also widely available and renewable. In recent years, researchers have applied low-cost biomass microfibers to lithium-ion battery separators. Cui et al. [61] used commercial photocopy paper (Xerox paper) as both the separator and mechanical substrate, and integrated all components of a lithium-ion battery through a simple lamination process onto this paper, creating a lithium-ion paper battery. In it, independent carbon nanotube thin films with high conductivity were employed as current collectors for both the anode and cathode, which showed a sheet resistance as low as ~5 Ω·m^−2^. Moreover, although this lithium-ion paper battery was thin (thickness: ~300 μm), it showed strong mechanical flexibility (bending capacity: less than 6 mm), as demonstrated in Figure 2. Furthermore, the battery could achieve a high energy density of 108 mAh·g^−1^. The accelerated development of flexible electronic devices has put forward higher requirements for lithium-ion batteries, requiring them to be not only safe but also flexible to adapt to integrated flexible electronic devices. Therefore, this lamination, through which all components are integrated into a lithium-ion paper battery, offers a new design concept for the manufacturing of flexible electronic devices.

Chen et al. [62] first used commercial rice paper (RP) as a separator for lithium-ion batteries. This separator is composed of cross-linked cellulose fibers with diameters ranging from 5 to 40 μm and its structure has a high porosity. This separator exhibits satisfactory electrochemical stability at a voltage of 4.5 V (vs. Li^+^/Li, measured in Li/RP/stainless-steel cells with 1 M LiPF_6_ in an ethylene carbonate/ethylene carbonate electrolyte) and shows good compatibility with electrodes such as graphite, LiFePO_4_, LiCoO_2_, and LiMn_2_O_4_. Compared with commercial PP/PE/PP separators, the RP separator has lower resistance under the same thickness conditions, which could be attributed to its highly porous structure. In addition, with its good flexibility, excellent electrochemical performance, and low cost, this RP separator is expected to partially replace the commercial separators currently used in low-power lithium-ion batteries.

**Figure 3:**
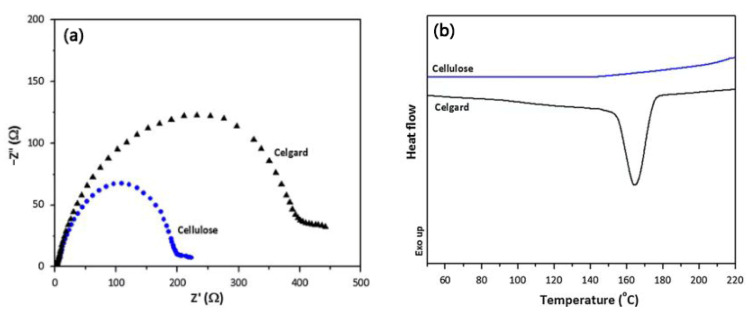
Electrochemical impedance spectra (**a**) and differential scanning calorimetry curves (**b**) of fibrous cellulose membrane and commercial PP separator [63].

Using commercial paper as the separator can successfully lead to lithium-ion batteries with excellent mechanical flexibility. This paper separator is lightweight and low-cost, but its thickness is high (Table 1), which is not conducive to reducing the internal resistance of lithium-ion batteries and increasing their energy density. A comparison of the properties of the various separators mentioned is shown in Table 1. To reduce the thickness of this separator and improve its pore structure, Alcoutlabi et al. [63] prepared a fibrous cellulose membrane via forcespinning using cellulose acetate as the raw material. When used as the separator for lithium-ion batteries, this membrane exhibits a three-dimensional network structure with a porosity of up to 76%, in which the cellulose fibers are randomly oriented and fully interconnected. This structure can not only enhance the electrolyte wettability and absorption capacity of the separator but also reduce the interface resistance between the separator and the electrode. Therefore, compared with commercially available PP separators, the fibrous cellulose membrane separator exhibits higher ion conductivity (2.12 × 10^−3^ S·cm^−1^), lower interfacial resistance (Figure 3a), and superior thermal stability, as can be seen from the differential scanning calorimetry results (Figure 3b). These advantages mean that a fibrous cellulose membrane can be used as the separator in high-performance lithium-ion batteries.

**Figure 4:**
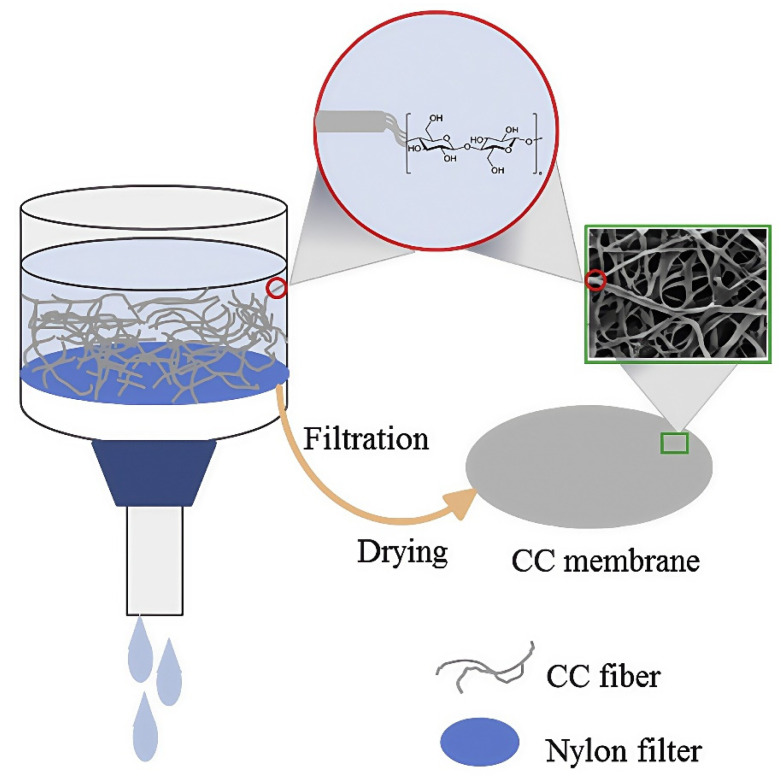
Schematic diagram of the preparation process of the Cladophora cellulose separator [64].

In addition to their usual thickness of over 60 μm, biomass microfibers have drawbacks such as large pore size and low mechanical strength, which can make it easier for the lithium dendrites to pierce the separator and cause battery short circuits [76]. To solve this problem, PAN et al. [64] prepared a lithium-ion battery separator using Cladophora cellulose (CC) as the raw material and adopted a process similar to that of papermaking, featuring vacuum filtration, as shown in Figure 4. The mesoporous CC separator has a thickness of ~35 μm and an average pore size of ~20 nm. Meanwhile, its Young’s modulus can reach up to 5.9 GPa. The decrease in separator thickness can reduce its volume resistance, and the nanoscale pore size provides it with high porosity. The excellent pore structure of the CC separator may be attributed to the high crystallinity of CC, which makes this type of separator less prone to aggregation when dried. Therefore, a LiFePO_4_/Li battery using the CC separator exhibits excellent cycling stability. After 50 cycles at a current density of 0.2 C, its discharge capacity retention rate can reach 99.5%.

Although multiple measures have been taken to address the issues of large pore size and low mechanical strength in cellulose microfiber-based separators, there is still a significant gap between them and current commercial separators. The surface of cellulose microfibers is rich in hydroxyl groups, which makes the separator highly flammable and hygroscopic. In recent years, various biomass microfiber-based composite separators have been prepared by combining biomass microfibers with functional substances. Compared to pure biomass microfiber-based separators, these composite separators have shown significantly superior electrochemical properties and safety performance [77,78,79].

### 3.2. Biomass Microfiber-Based Composite Separators

**Figure 5:**
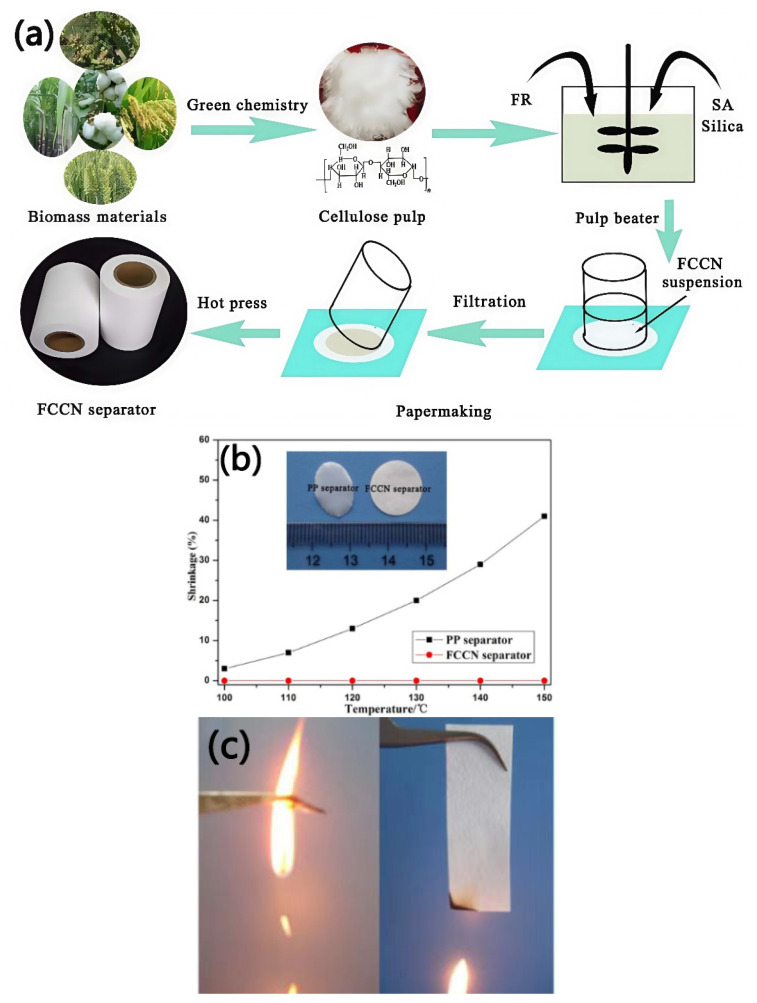
Schematic diagram of the manufacturing process of the FCCN separator (**a**). Thermal shrinkage rate (**b**) (the inserted photograph shows the two separators after being heated at 150 °C for 0.5 h) and combustion behavior (**c**) of the PP separator and FCCN separator [65].

Cui et al. [65] first fabricated a cellulose-based composite nonwoven separator (FCCN separator) using cellulose fibers and flame retardants as raw materials, as shown in Figure 5. In comparison with commercial PP separators, the FCCN separator exhibits better rate performance, which could be ascribed to its interconnected pore spaces and hydrophilic properties, resulting in high electrolyte absorption and ion conductivity. In addition, the FCCN separator also exhibits superior thermal stability, which may be due to the outstanding heat resistance of cellulose. When used for a LiFePO_4_/Li half-cell at a high temperature of 120 °C, the FCCN separator exhibits excellent cycling performance, while the PP separator is almost unable to charge and discharge normally. Furthermore, the higher limiting oxygen index (LOI) value of the FCCN separator indicates its superior flame-retardant properties compared to the PP separator. Good heat resistance and flame retardancy help enhance the safety of lithium-ion batteries, while the simple papermaking process enables the large-scale production and application of this composite separator.

**Figure 6:**
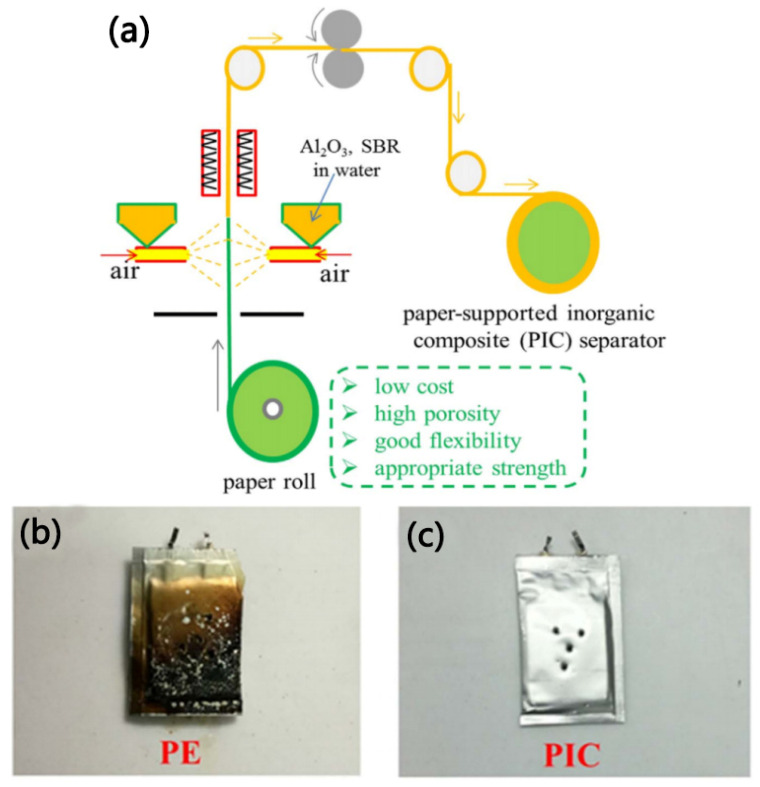
Schematic diagram of the preparation process of the PIC separator (**a**). The nail penetration test results of pouch cells with different separators (the PE separator (**b**) and the PIC separator (**c**), respectively) [66].

Wang et al. [66] prepared a paper-based inorganic composite separator (PIC separator) by spraying Al_2_O_3_ particles onto the surface of commercial paper-based material, as shown in Figure 6a. Due to the interaction between the hydroxyl groups of the paper-based material and the polar groups of Al_2_O_3_, the adhesion between the paper-based material and Al_2_O_3_ is enhanced, thereby ensuring the structural stability of the PIC separator. Compared with traditional PE separators (43%), the PIC separator has better porosity (56%). This could be due to the fact that after spraying Al_2_O_3_ particles, the large pores of the paper-based structure are partially covered and transformed into mesopores or micropores, thereby improving their absorption of electrolytes and reducing the self-discharge of lithium-ion batteries. Moreover, in the nail penetration experiment of batteries, as shown in Figure 6b,c, when the nail penetrated the bag battery featuring the PIC separator, no smoke or combustion was observed, while when the bag battery containing the PE separator was penetrated by the nail, a burning phenomenon was observed on the surface. This could be attributed to the better thermal stability of the cellulose fibers and the Al_2_O_3_ coating, which contribute to the PIC separator’s satisfactory safety performance for use in lithium-ion batteries.

### 3.3. Biomass Nanofiber-Based Separators

Although there have been reports of biomass microfiber-based separators used as lithium-ion battery separators [80,81,82], their pore structure and mechanical properties still have inherent defects which limit their practical applications. Compared with biomass microfibers, biomass nanofibers with a nanometer-scale diameter (cellulose acetate membrane (fiber diameter: 1.18 μm) [63] vs. CNFs (fiber diameter: <50 nm) [79]) and a micrometer-scale length, as well as better mechanical properties and thermal stability, are more suitable for use as separators in lithium-ion batteries [83,84]. Among them, the nanometer-scale diameter is a key factor in controlling the pore structure of the separators.

**Figure 7:**
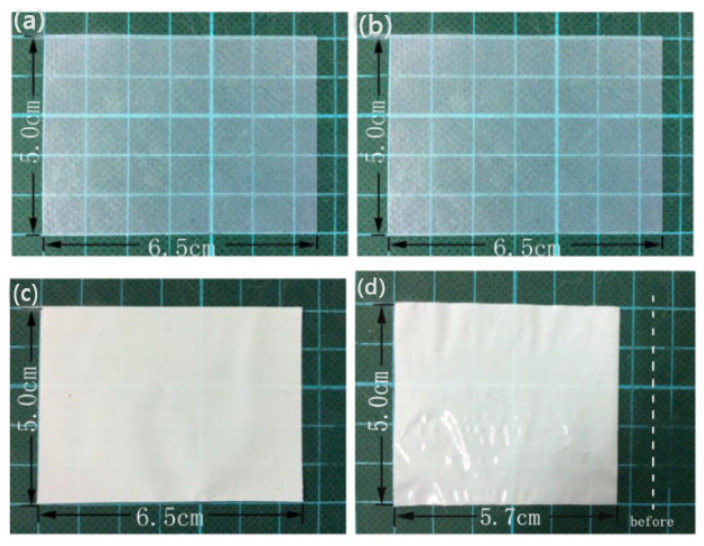
Photographs of BC separators (**a**,**b**) and Celgard^®^ separators (**c**,**d**) before (**a**,**c**) and after (**b**,**d**) annealing at 100 °C for 12 h [67].

Lee et al. [85] first employed a simple self-assembly method to transform a cellulose nanofiber (CNF) suspension into cellulose nanofiber paper. The obtained cellulose nanofiber paper has a unique pore structure, and it can also be adjusted by changing the proportion of different solvents (isopropanol/water) in the CNF suspension, ultimately forming abundant network channels and displaying excellent mechanical strength. However, relying solely on adjusting the solvents is not sufficient to control the pore structure of the separator properly. Jiang et al. [67] used a bacterial cellulose (BC) nanofiber separator in a lithium-ion battery. Unlike ordinary cellulose, BC fibers have an average diameter of less than 100 nm and can form a porous three-dimensional structure through mutual cross-linking and overlapping, which enables the BC separator to have good electrolyte wettability. In addition, compared with commercial Celgard^®^ separators, the BC separator has outstanding thermal stability. After heat treatment at 100 °C for 12 h, the BC separator showed almost no thermal shrinkage, while the commercial Celgard^®^ separator rapidly contracted in the first 10 min, as shown in Figure 7. Even after heat treatment at a high temperature of 180 °C for 3 h, the BC separator maintained good structural stability, while the Celgard^®^ separator rapidly melted at 165 °C. However, despite its many advantages, the ion conductivity of the BC separator is not satisfactory and further improvements are still needed to enhance this characteristic.

In order to improve the pore structure of BC separator, Xu et al. [68] prepared a 2,2,6,6-tetramethylpiperidine-1-oxyl (TEMPO)-oxidized bacterial cellulose (TOBC) separator by first converting the BC separator into a BC suspension using 2,2,6,6-tetramethylpylperidine-l-oxyl radial (TEMPO)-mediated oxidation, followed by vacuum filtration. The TOBC separator is composed of fibers with an average diameter of ~48 nm, which can be attributed to the change in the chemical structure of BC fibers caused by TEMPO oxidation. The mutual repulsion between aldehyde and aldehyde groups in the BC fibers enhances their dispersibility in water, thereby improving the uniformity of pore size distribution of the TOBC separator after drying. The TOBC separator has high porosity (91.1%), a good electrolyte absorption capacity (339%), low interface resistance with a lithium electrode (96 Ω), and an ion conductivity up to 13.45 mS·cm^−1^. When the TOBC separator is used for Li/LiFePO_4_ half-cells, the discharge specific capacity of the battery can reach 166 mAh·g^−1^ at a current density of 0.2 C, and the capacity retention rate achieves 94% after 100 cycles.

**Figure 8:**
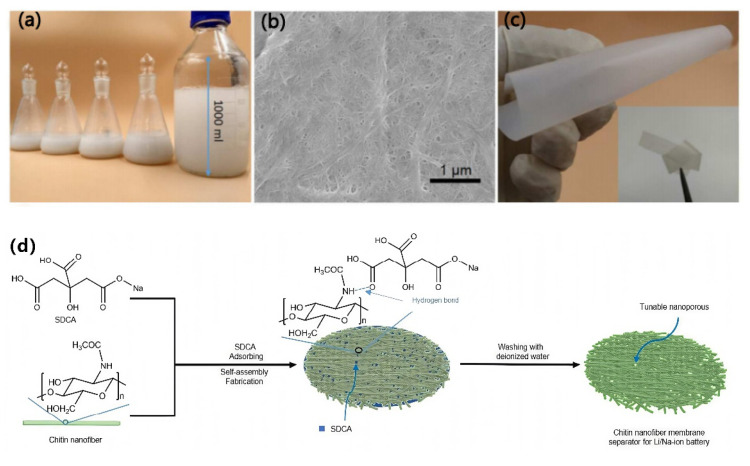
(**a**) Photograph of chitin nanofiber suspension. (**b**) Scanning electron microscope (SEM) image of chitin nanofibers. (**c**) Photograph of CNM obtained from chitin nanofiber suspension by vacuum drying. (**d**) Schematic diagram of manufacturing process of CNM separator using SDCA [69].

In addition to the oxidation method, the template method is also an effective way to create pores during the production of a separator. Yu et al. [69] prepared porous chitosan nanofiber membrane (CNM) separators using sodium dihydrogen citrate (SDCA) as a template, as shown in Figure 8. The hydroxyl groups of SDCA can form hydrogen bonds with the functional groups of chitosan nanofibers, allowing SDCA to be uniformly adsorbed on the surface of chitosan nanofibers. During solvent evaporation, SDCA plays a role in preventing the tight packing of chitosan nanofibers. After removal of SDCA via washing, a porous CNM separator is obtained, and its pore structure can be regulated by changing the mass ratio of SDCA to chitin nanofibers. The electrochemical performance of the prepared Chitin/SDCA-40% separator at room temperature is comparable to that of a commercial PP separator. However, under a high-temperature condition of 120 °C, it exhibits a significantly superior electrochemical performance.

### 3.4. Biomass Nanofiber-Based Composite Separators

**Figure 9:**
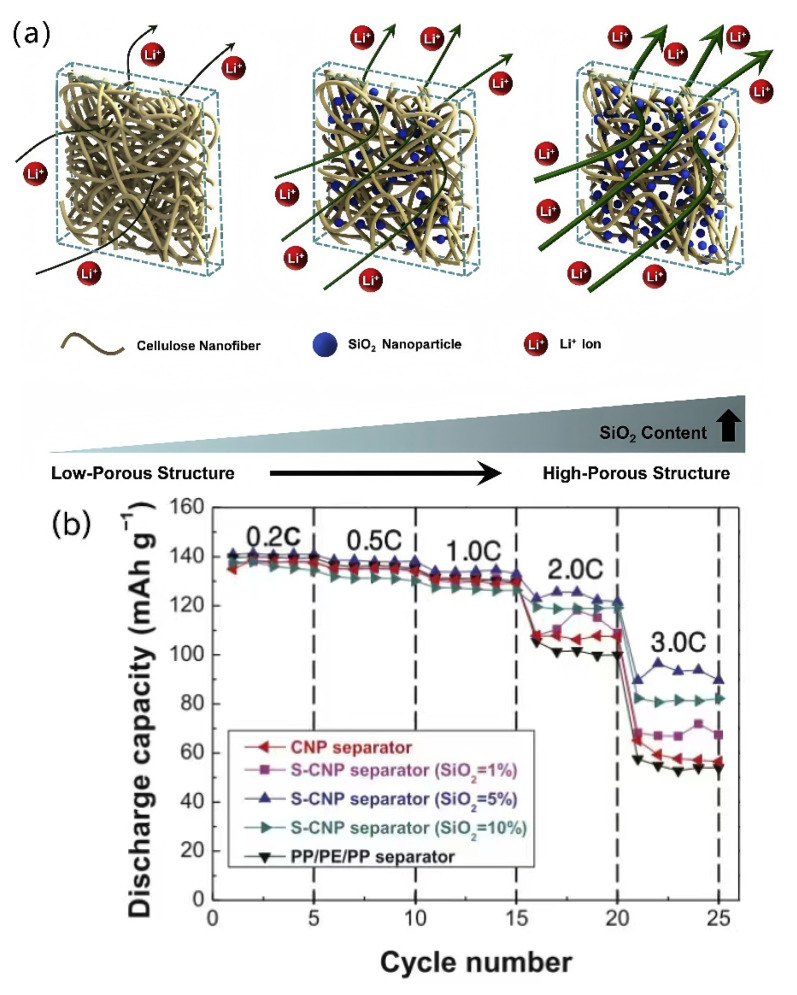
(**a**) Schematic diagram of S-CNP separator and influence of SiO_2_ nanoparticles on its ionic transport. (**b**) Rate performance of various S-CNP separators and other separators [70].

Compared to biomass nanofiber-based separators, biomass nanofiber-based composite separators exhibit superior performance, such as superior porosity and liquid absorption, and have become an important development direction [86,87,88]. Lee et al. [70] added colloidal SiO_2_ nanoparticles, a pore-forming agent, to a cellulose nanofiber (CNF) suspension and synthesized a SiO_2_-containing cellulose nanofiber paper separator (S-CNP separator) by vacuum filtration. SiO_2_ nanoparticles can prevent tight packing between nanofibers during solvent evaporation, thus effectively improving the porosity of the cellulose nanofiber-based separator. More importantly, the pore structure of the prepared S-CNP separator can be adjusted according to the SiO_2_ content in the suspension, as shown in Figure 9. The experimental results showed that the S-CNP separator containing 5 wt.% SiO_2_ exhibited the best ion conductivity and electrochemical performance, and its rate performance was better than those of the CNP separator without SiO_2_ and traditional commercial separators.

**Figure 10:**
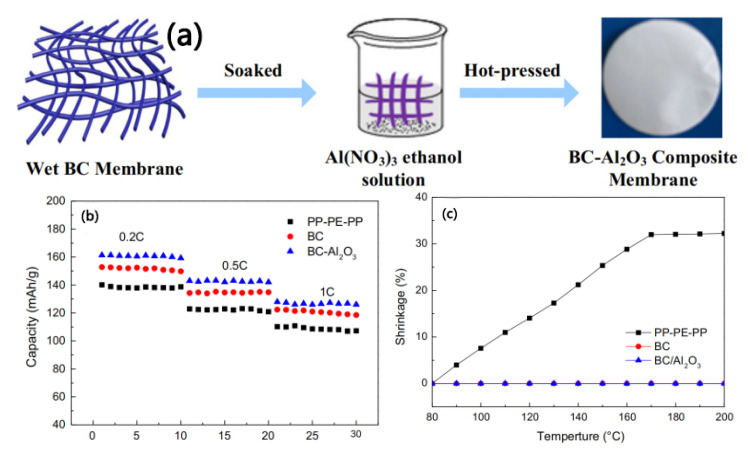
(**a**) Schematic diagram of manufacturing process of BC-Al_2_O_3_ composite separator. (**b**) Rate performance of different separators. (**c**) Thermal shrinkage behavior of different separators at different temperatures for 30 min [71].

Although the S-CNP separator prepared by Lee et al. has excellent electrochemical performance, its complex preparation method is not conducive to large-scale production. In view of this, Xu et al. [71] prepared BC/Al_2_O_3_ nanofiber composite separators using a simple in situ pyrolysis method, as shown in Figure 10a. In high-temperature air, the Al(NO_3_)_3_ coated on the surface of BC decomposes and releases NO_2_ and O_2_ molecules, and the formed Al_2_O_3_ can be covalently connected to BC fibers. The Al_2_O_3_ on the surface of BC fibers helps to improve the dispersion of BC fibers, thereby increasing the porosity of the prepared separator. The test results show that the BC/Al_2_O_3_ composite separator has high porosity (74.7%), excellent electrolyte absorption (625%), and outstanding ion conductivity (4.91 mS·cm^−1^). Compared with the BC separator and a commercial PP-PE-PP separator, the BC/Al_2_O_3_ composite separator exhibits superior rate performance in batteries, as shown in Figure 10b. It is particularly noteworthy from Figure 10c that the BC/Al_2_O_3_ composite separator possesses remarkable thermal stability at a high temperature of 200 °C, while the commercial PP-PE-PP separator shows significant thermal shrinkage under the same conditions.

**Figure 11:**
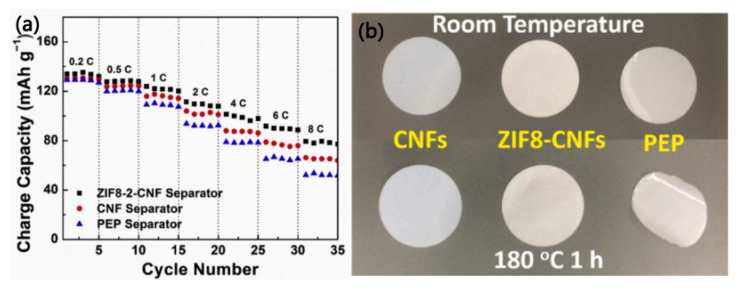
(**a**) Rate performance of lithium-ion batteries with different separators. (**b**) Thermal shrinkage behavior of different separators at 180 °C for 1 h [72].

Wu et al. [72] first introduced zeolitic imidazolate framework-8 (ZIF-8) into the separator of lithium-ion batteries. A ZIF-8-CNF composite separator was fabricated by synthesizing ZIF-8 crystals on the surface of cellulose nanofibers (CNFs), which helps to prevent CNF aggregation and thereby improves their pore size uniformity and electrolyte wettability. Compared to the pure CNF separator (porosity: 42%), the porosity of the ZIF8-2-CNF (ZIF8:CNF ratio: 0.6:1) separator can be increased to 55%. Moreover, compared with the pure CNF separator and a commercial PEP separator, the ZIF8-2-CNF separator shows superior electrochemical performance in batteries and better thermal stability under a high temperature condition of 180 °C, as can be seen in Figure 11a,b, respectively. It is worth noting that in lithium-ion batteries, no obvious decomposition of the main components was observed for the ZIF8-2-CNF and CNF separators before 4.4 V, comparable to the commercial PEP separator.

**Figure 12:**
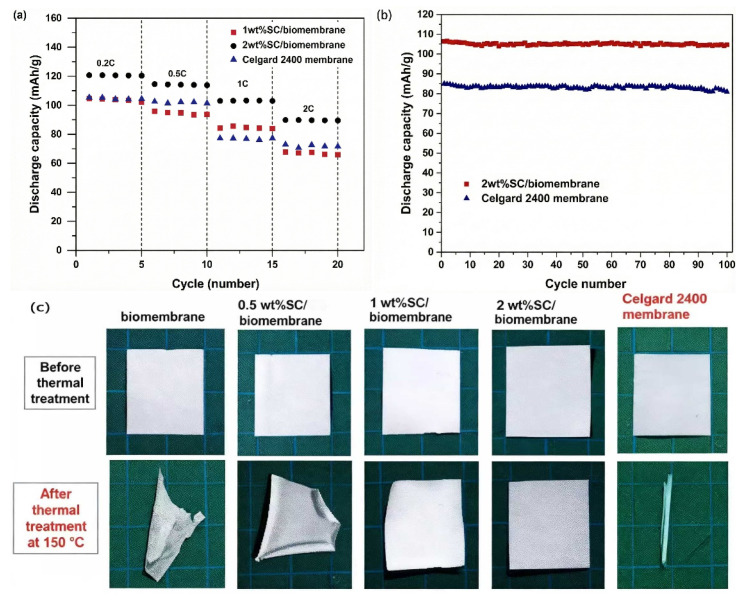
(**a**) Rate performance of lithium-ion batteries with different separators. (**b**) Cycling performance of lithium-ion batteries with different separators at 1 C. (**c**) Thermal shrinkage behavior of different separators after heat treatment at 150 °C [73].

As is well known, the thermal stability of the separator plays a decisive role in the high-temperature performance of the battery. Manuspiya et al. [73] used sulfonated cellulose (SC) and poly(lactic acid)/poly(butylene succinate) (PLA/PBS) composites to prepare SC/biomembranes by a phase inversion method. The test results revealed that the 2 wt% SC/biomembrane exhibited the best electrochemical performance. Its porosity was estimated to be 87.7%, significantly higher than that (42.1%) of the commercial Celgard 2400 membrane, which resulted in its better electrolyte uptake and lower interfacial resistance with electrodes. When used as a separator in a LiCoO_2_/graphite battery, a discharge specific capacity of 106 mAh·g^−1^ was delivered by the battery assembled with the 2 wt% SC/biomembrane after 100 cycles at 1 C, while the battery with the Celgard 2400 membrane displayed a capacity of only 81 mAh·g^−1^, as shown in Figure 12a,b. In addition, the 2 wt% SC/biomembrane has superior thermal stability, with almost no thermal shrinkage at a high temperature of 150 °C, while the Celgard 2400 membrane exhibited significant severe deformation, as seen in Figure 12c. Therefore, the 2 wt% SC/biomembrane is more promising for use as a separator in high-safety lithium-ion batteries.

**Figure 13:**
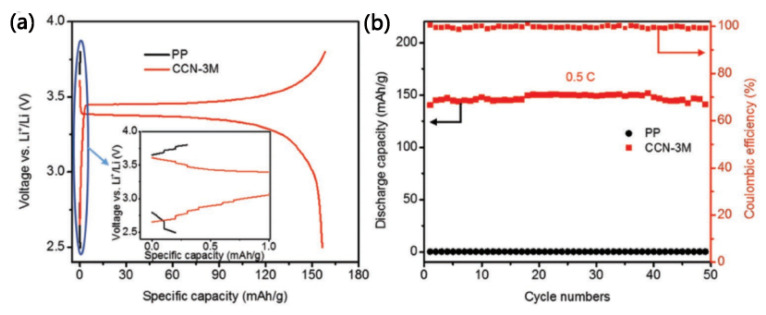
(**a**) The charge–discharge voltage profiles of LiFePO_4_/Li half-cells with different separators at 0.5 C (120 °C). The figure insert shows the failure of the cell with the PP separator. (**b**) Cycling ability of the cells with different separators at 0.5 C (120 °C) [74].

Yao et al. [74] used natural polymer nanofibers (chitosan nanofibers) as raw materials and chemically modified them by grafting cyanoethyl groups onto their surfaces to obtain a dense chitin nanofiber-based separator (CCN separator). The CCN separator possesses an extremely high tensile strength (120 MPa), far exceeding the porous chitosan nanofiber-based separator (80 MPa) previously reported. Moreover, the grafting of cyanoethyl groups gives CCN separators high ion conductivity (0.45 mS·cm^−1^), which is approximately 13 times that of the unmodified chitosan nanofiber-based separators (0.035 mS·cm^−1^). Furthermore, the excellent thermal stability of CCN separators enables LiFePO_4_/Li batteries to operate at 120 °C, which is not achievable with commercial polyolefin separators, as shown in Figure 13. The chemical modification method for chitosan nanofibers presented in this study could provide inspiration for the preparation of other natural polymer fiber-based composite separators.

**Figure 14:**
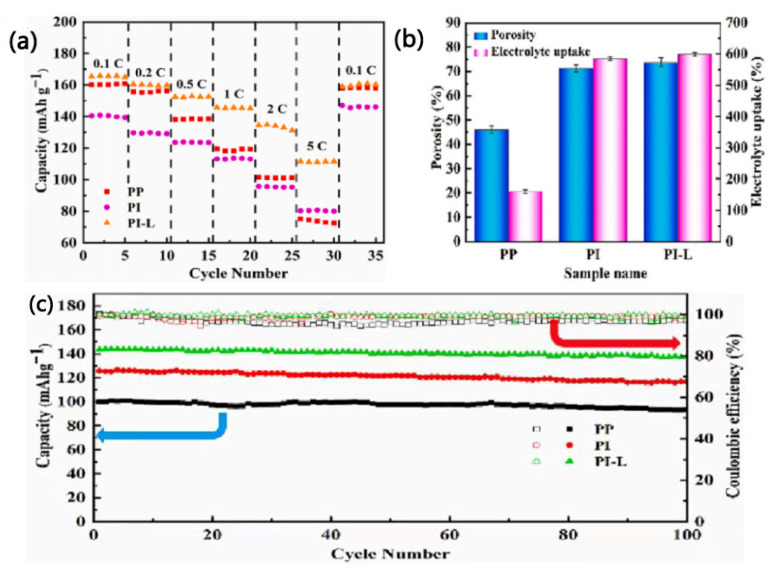
(**a**) Rate performance of batteries with different separators at room temperature. (**b**) Porosity and electrolyte uptake of different separators. (**c**) Cycling performance of batteries with different separators at 1 C [75].

Wang et al. [75] prepared a new biomass nanofiber separator (PI-L separator) by using polyimide (PI) and lignin (L) as raw materials through simple physical mixing and electrospinning techniques. In addition to the excellent thermal stability of the PI-L separator (above 450 °C), the polar groups of lignin can also enhance the affinity between the PI-L separator and the electrolyte, thereby increasing the electrolyte absorption of the PI-L separator (592%) (Figure 14b). The PI-L separator thus exhibits high ion conductivity (1.78 × 10^−3^ S·cm^−1^) and lithium ion mobility (0.787). Additionally, due to the introduction of lignin, the cycling and rate performance of the battery are improved, as shown in Figure 14a. After 100 cycles at the 1 C rate, the capacity retention rate of the LiFePO_4_|PI-L|Li battery reaches 95.1%, which is clearly higher than those of PI separators and current commercial PP separators (90%) (Figure 14c). Moreover, the SEM results indicate that lignin can inhibit the formation of lithium dendrites and generate a stable solid electrolyte interphase (SEI) film, which helps to enhance the safety of the PI-L separator [89].

## 4. Summary

In recent years, great progress has been made in the application of biomass fiber-based separators in lithium-ion batteries due to their excellent electrolyte compatibility, thermal stability, abundant reserves, and environmental sustainability. As well as summarizing the advantages of biomass fiber-based separators, their shortcomings, such as pore structure, mechanical properties, and industrialization issues, are also pointed out in this review. This review will be beneficial for researchers, providing a reference for urgent issues to start from when preparing suitable separators for high-performance lithium-ion batteries.

(i) In the battery industry, to prevent lithium dendrites from piercing the separator, the pore size of the separator should generally be less than 1 µm. Therefore, the pore structure of the separator must be practical: the pore size needs to be controlled at the nanometer level and the pore size distribution needs to be uniform, as this is beneficial for the rapid migration of lithium ions within the separator.
(ii) Appropriate modification of biomass fibers can improve their physicochemical properties, allowing researchers to fully utilize the advantages of biomass fiber-based separators while avoiding the impact of their disadvantages. Especially for synthesizing biomass fiber-based composite separators, the addition of other substances can effectively alleviate fiber aggregation and then improve the electrolyte absorption and mechanical strength of the separators.
(iii) The mass production of biomass fiber-based separators for lithium-ion batteries has not yet been achieved. This indicates that their preparation process and production equipment do not currently meet the requirements for large-scale production. Therefore, research efforts can be increased in these two areas, for example, to improve textile and surface coating technologies.

## Figures and Tables

**Table 1:** The characteristics of the various separators mentioned in the manuscript.

Separator Type	Thickness (μm)	Porosity (%)	Average Pore Size/Range (nm)	Thermal Stability (°C)	Liquid Absorption (%)	Ionic Conductivity (S·cm^−1^)	Tensile Strength (MPa)	Ref
Commercial Xerox paper	~100	-	-	-	-	-	-	[61]
Commercial rice paper	~100	-	-	90	-	-	-	[62]
Fibrous cellulose membrane	50	76	-	180	370	2.12 × 10^−3^	-	[63]
CC	35	46	20/2~200	150	Higher than Solupor^®^	0.4 × 10^−3^	-	[64]
FCCN	40	70	-/100~200	300	270	2 × 10^−3^	45	[65]
PIC	48	56	-	130	Higher than PE	1.64 × 10^−3^	-	[66]
BC	10–15	-	-	180	Higher than Celgard^®^ 2325	-	78	[67]
TOBC	29	91.1	-	200	339	13.45 × 10^−3^	97	[68]
CNM	25	54	-/10–150	150	242	0.064 × 10^−3^	83	[69]
S-CNP	32	48	-	150	Higher than PP/PE/PP	2.97 × 10^−3^	-	[70]
BC-Al_2_O_3_	30	74.7	-	200	625	4.91 × 10^−3^	140	[71]
ZIF8-CNF	32	55	-/400–650	200	Higher than PEP	1.41 × 10^−3^	-	[72]
SC/biomembrane	-	87.7	-	150	290.6	3.24 × 10^−3^	-	[73]
CCN	12	5.4	3–5	170	Higher than PP	0.45 × 10^−3^	120	[74]
PI-L	-	-	-	160	592	1.78 × 10^−3^	-	[75]
PP	25	55	-/100–500	Melting: 165	125	0.65 × 10^−3^	12	[65]

## Data Availability

Data sharing is not applicable.
